# Open datasets wanted for tracking the insect decline: let’s start from saproxylic beetles

**DOI:** 10.3897/BDJ.9.e72741

**Published:** 2021-11-02

**Authors:** Alessandro Campanaro, Francesco Parisi

**Affiliations:** 1 Research Centre for Plant Protection and Certification, Council for Agricultural Research and Economics, Firenze, Italy Research Centre for Plant Protection and Certification, Council for Agricultural Research and Economics Firenze Italy; 2 GeoLAB - Laboratory of Forest Geomatics, Department of Agriculture, Food, Environment and Forestry, Università degli Studi di Firenze, Firenze, Italy GeoLAB - Laboratory of Forest Geomatics, Department of Agriculture, Food, Environment and Forestry, Università degli Studi di Firenze Firenze Italy

**Keywords:** biodiversity crisis, datasets, forest ecosystems, insect communities, Zenodo repository

## Abstract

**Background:**

We present six datasets of saproxylic beetles collected between 2012 and 2018 in Central and Southern Italian forests. Saproxylics represent one of the main components in forest ecosystems in terms of diversity, species richness and functional traits and, for this reason, they are an important target group for studying the modification of forests over time. The datasets consist of annotated checklists and were published on Zenodo repository.

**New information:**

Overall, 1,171 records are published, corresponding to 918 taxa (taxonomy at species or subspecies level). The taxa are scarcely shared amongst the areas, 80.2% of them are exclusive, indicating that the beetle communities are substantially different. In consideration of the biodiversity crisis we are passing through, which is especially dramatic for the insects, we want to promotecollaboration amongst researchers for making datasets available in open repositories. This will improve the possibility for researchers and forest managers of analysing the state of species distribution that could serve for long-term studies on the variation of insect communities. We encourage repeating species assessment in the same localities in order to evaluate the trends in insect communities over time and space.

## Introduction

 The insect decline is one of the most crucial biological crises we are facing in the Anthropocene (Wagner et al. 2021).

Insects are affected by a global decline in terms of individual abundance as demonstrated by much experimental evidence: a change in aerial insect biomass in outhern Britain have been analysed over 31 years by Shortall et al. (2009); Hallmann et al. (2017) registered a decline of 76% in flying insect biomass over 27 years in protected nature areas in Germany; Lister and Garcia (2018), comparing arthropod biomass captured in 1976 with samples of 2011, 2012 and 2013 in a rainforest in Puerto Rico, found values from 4 to 8 times lower.

The decrease in insect species number has also been the object of several studies: Forister et al. (2010) analysed 30 years of butterfly presence-absence data in western North America and found a decline of richness at the lowest-elevation sites; Biesmeijer et al. (2006) found a significant fall in diversity in Britain and The Netherlands pre-versus post-1980 in a study on the insect extinction determined by urbanisation; Fattorini (2011) calculated an impressive decline of species amongst butterflies (i.e. *Cacyreusmarshalli*), tenebrionid beetles (i.e. *Alphitophagusbifasciatus*, *Gnatoceruscornutus*, *Latheticusoryzae*, *Triboliumcastaneum*, *Triboliumconfusum* and A*lphitobius diaperinus*) and scarabaeoids from 1885 to 1999, even if with different patterns; over two decades, the European Butterfly Indicator shows a dramatic loss of grassland biodiversity (half of the species has declined) (Van Swaay et al. 2010).

In their comprehensive review, Sanchez-Bayo and Wyckhuys 2019 have estimated that the current proportion of insect species in decline (41%) is twice as high as that of vertebrates and the pace of local species extinction (10%) is eight times higher. Insects are facing an unprecedented decline, which has globally reached alarming proportions, especially in the last two decades: even a third of the considered beetle species are at risk of extinction and almost half of studied bees and ants is threatened (Sanchez-Bayo and Wyckhuys 2019).

mportant evidence of the insect decline comes from the number of threatened species reported by the IUCN European Red Lists (Table 1) that ranges from 9% to 26% as a proportion of the total number of species assessed at European level for taxonomic groups. These documents provide other significant data, the high number of “data deficient” records (i.e. species whose sufficient knowledge in terms of life cycles and ecological traits is too poor and there were no assessments according with the IUCN criteria) indicating that decline of the most common species is generally neglected. In some cases (i.e. bees, Andrenidae, Apidae, Colletidae, Halictidae, Megachilidae, Melittidae families), for more than half of the species, the available data are not sufficient (Table 1). Globally, of more than 1 million of the estimated number of species, only 0.9% have yet been evaluated by IUCN.

Europe is aware of this phenomenon; the EU Biodiversity Strategy for 2030 (Brussels, 20.5.2020 COM(2020) 380 final) highlights the alarming decline of insects, particularly pollinators and their role as key indicators of the health of agroecosystems. It states the full implementation of the EU Pollinators initiative (Brussels, 1.6.2018 COM (2018) 395 final) and the reversion of the decline in pollinators by 2030.

To make the strategy effective, an analysis of drivers for the insect decline (Sanchez-Bayo and Wyckhuys 2019) should be associated with long term monitoring plans and collection of diversity data. At the moment, very few standardised datasets are available for long-term analysis and spatial records of insects are very scarce worldwide (Rocha-Ortega et al. 2021). Our data paper proposes to answer the call of “urgent need of data”, which is particularly needed for insects with no economic value, launched by Wagner (2020) and to the appeal launched by Cardoso et al. (2020), for urgent actions towards insect extinctions which includes the necessity to fill many gaps in the knowledge of species presence and distribution. Moreover, we encourage using our datasets as point “0” for further investigations.

In this paper, we focus on the communities of beetles inhabiting the Italian forests. Amongst them, the saproxylics represent one of the main components, in terms of diversity, species richness and functional traits (Parisi et al. 2018, Seibold et al. 2021, Thorn et al. 2020). Many species of saproxylics in the adult phase also play a role as pollinators: 16% of the saproxylics of England, Wales and Scotland (Falk 2021). Even though many research groups are actively involved in analysing the saproxylic fauna and many researchers have been published in the fields of taxonomy, systematics and ecology, nature conservation and checklist of species for the Italian forests are scarcely available. It results in the impossibility of tracking the modification of insect communities over time.

To start the job in this direction, we published the datasets of saproxylic species occurrence coming from field experiments carried out between 2012 and 2018 in central and southern Italian forests.

## General description

### Purpose

We wish to promote collaboration amongst researchers for making datasets on saproxylic beetles available in open repositories. This will improve the possibility for researchers and forest managers of analysing the state of species distribution and presence that could serve for long term studies on the variation of insect communities.

## Sampling methods

### Study extent

**Figure 1.**
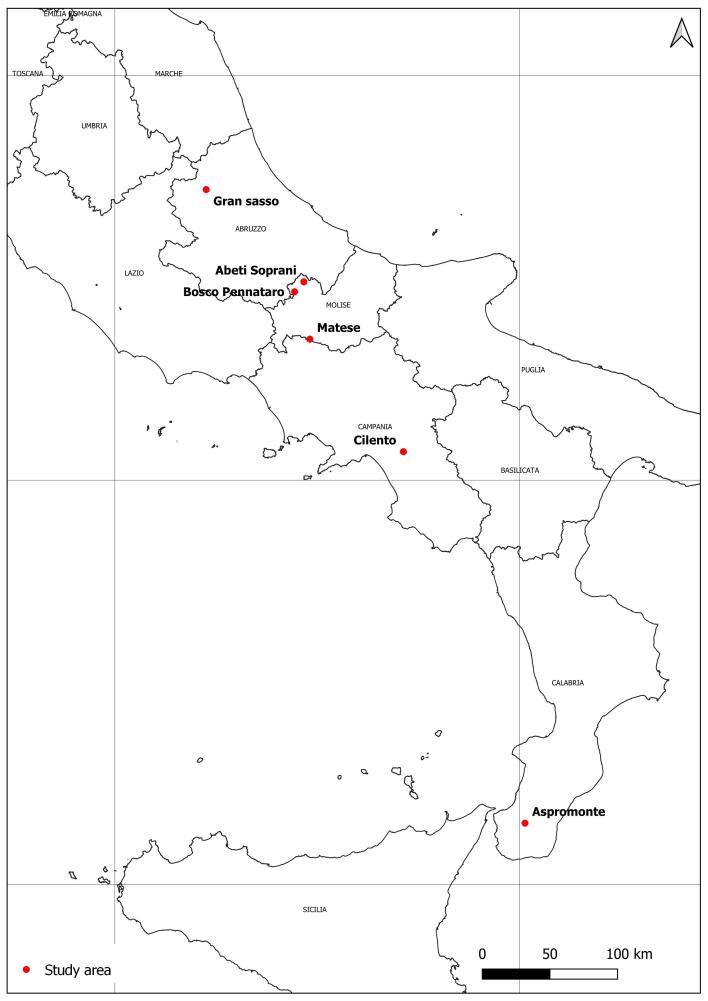
Map of the study sites located in central and southern Italy.

The datasets include information of species collected in six forested sites of the Apennines, corresponding to 15 plots (Fig. 1).

**Figure 2.**
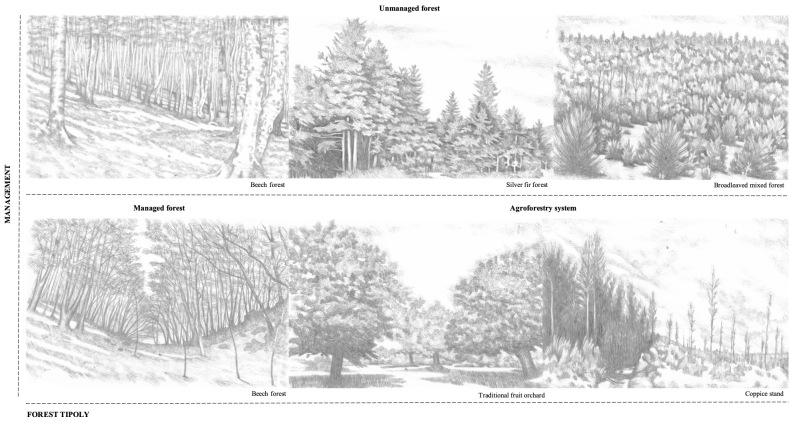
A graphical representation of forest ecosystems studied (drawings by G. Parisi).

Forest typologies fall into five EUNIS habitat types (European Environmental Agency, EUNIS Habitat Classification 2017): *Fagus* forest on non-acid soils, Southern Apennine *Abiesalba* forests, Thermophilous deciduous woodland, southern Italian *Fagus* forests, Coppice and early-stage plantations. Forest areas also differ for the actual and historical management: from uneven unmanaged forests, coppice stands to traditional orchards (Fig. 2). Some of the study areas are included in Natura 2000 sites, National Parks or UNESCO sites (Table 2). A detailed description of the study areas is reported in research papers that have been published on the basis of these datasets (Parisi et al. 2016, Parisi et al. 2019, Parisi et al. 2020a, Parisi et al. 2020b, [Bibr B7382619][Bibr B7382649] ,Zanetti and Parisi 2019).

## Geographic coverage

### Description

The study covers forests sited in central and southern Italy along a latitudinal gradient (Fig. 1).

### Coordinates

38.18 S and 42.5096 N Latitude; 13.5679 W and 15.784167 E Longitude.

## Collection data

### Collection name

The insects were collected using flight interception traps and emergence traps from 2012 to 2018. Flight interception traps are made by transparent panes of 60x40 cm and they were left active from May to September and checked with a periodicity of 30 days. Emergence traps are made by breathable plastic bags enveloping a portion of deadwood (1 m long) and were connected to a jar for the collection of emerging insects. Materials have been preserved in alcohol (70%) and then mounted (on cards and pinned) and dried for identification at species level. The taxomomy of the species (Family, Scientific Name and Authorship) were provided by the entomology specialists (see Acknowledgements). All the scientific names and authorships have been, subsequently, validated and harmonised following the Fauna Europea Database. The entomological material is preserved at the Department of Agriculture, Food, Environment and Forestry (DAGRI) - University of Florence.

## Usage licence

### Usage licence

Creative Commons Public Domain Waiver (CC-Zero)

## Data resources

### Data package title

Annotated checklists of beetles (Insecta, Coleoptera)

### Number of data sets

1

### Data set 1.

#### Data set name

Annotated checklists of beetles (Insecta, Coleoptera)

#### Number of columns

1

#### Description

The datasets consist of annotated checklists of beetles (Insecta, Coleoptera) and were published on Zenodo repository (Table 3). Overall, 1,171 records are published, corresponding to 918 taxa (taxonomy at species or subspecies level). The taxa are scarcely shared amongst the areas, 80.2% of them are exclusive, indicating that the beetle communities are substantially different. The fields used for the datasets follows the Darwin Core vocabulary (Darwin Core Maintenance), the list of used terms and their adoption for the information recorded in our datasets are described below: catalogNumber (a unique identifier number associated with the biological entity in that catalogue), order, family, scientificName (genus species or genus sp. or genus species subspecies of the biological entity), scientificNameAuthorship, individualCount (total number of the individuals sampled), measurementDeterminedDate (year(s) of sampling), Country, decimalLatitude, decimalLongitude, geodeticDatum, locality, habitat (habitat type according to EUNIS habitat classification 2017), samplingProtocol, recordedBy, identifiedBy, dynamicProperties (IUCN Red List categories), associatedReferences, scientificNameID (the unique identifier for the species, if the specific names does not match with any name in the Fauna Europea Database, the field is NA and the scientificName reported corresponds to the name indicated by the entomology specialist) and taxonID. For datasets of the study area Gran Sasso, Cilento and Aspromonte, the fields decimalLatitude, decimalLongitude, geodeticDatum are not provided; the geographic coordinates are described in the Upload Description.

Datasets are in the form of .csv files.

In the published papers which refer to these datasets (Table 3), the trophic categories for the saproxylic species are reported, while analysing of the trophic interactions have been considered in Parisi et al. (2019) and Parisi et al. (2020c), giving the opportunity, if the survey is to be repeated, to obtain information on the change of ecological network as suggested by Petsopoulus et al. (2021).

**Data set 1.** 

Column label	Column description
Records	Zenodo repository

## Figures and Tables

**Table 1.** Number in % of threatened species reported by the IUCN European Red Lists.

Insect group	Threatened species all Europe, %	Threatened species EU27/28 level, %	Data deficient all Europe, %	Data deficient EU27/28 level, %	Reference
Saproxylic beetles	17.9	21.7	24.4	20.4	Calix et al. (2018)
Grasshoppers, crickets and bush-crickets	25.7	28	10	8.5	Hochkirch et al. (2016)
Dragonflies	15	16.4	3.6	2.2	Kalkman et al. (2010)
Bees	9.2	9.1	56.7	55.6	Nieto et al. (2014)
Butterflies	9	7	0.9	0.9	Van Swaay et al. (2010)
Insect group	Threatened species		Data deficient		Reference
Mediterranean butterflies	5		6		Numa et al. (2016)

**Table 2.** Annotated list of the study sites from which the data originated. ^1^Datum: WGS84.

Study sites	Sampling site	No. of plots	Sampling year	Natura 2000 site	Protected area	Lat^1^	Long^1^	EUNIS Habitat type	Forest management
Gran Sasso	Incodara	1	2013, 2016	IT7110202 “Gran Sasso”	Gran Sasso e Monti della Laga National Park	42.5123	13.4735	*Fagus* forest on non-acid soils	Unmanaged forest
Gran Sasso	Prati di Tivo	1	2013, 2016	IT7110202 “Gran Sasso”	Gran Sasso e Monti della Laga National Park	42.5096	15.5679	*Fagus* forest on non-acid soils	Unmanaged forest
Gran Sasso	Venacquaro	1	2013, 2016	IT7110202 “Gran Sasso”	Gran Sasso e Monti della Laga National Park	42.4988	13.5139	*Fagus* forest on non-acid soils	Unmanaged forest
Abeti Soprani	Abeti Soprani	1	2012 and 2013			41.8608	14.2936	Southern Apennine *Abiesalba* forests	Unmanaged forest
Bosco Pennataro	Bosco Pennataro	1	2014 and 2015		UNESCO "Collemeluccio-Montedimezzo-Alto Molise"	41.7489	14.1972	Thermophilous deciduous woodland	Unmanaged forest
Matese	Matese	4	2018	IT7222287 “La Gallinola - Monte Miletto - Monti del Matese”	Matese National Park	41.4522	14.3503	Southern Italian *Fagus* forests	Managed forest
Cilento	Monti Alburni	1	2013, 2016	IT8050033 “Monti Alburni”	Cilento, Vallo di Diano e Alburni National Park	40.5136	15.3292	*Fagus* forest on non-acid soils	Unmanaged forest
Cilento	Monti Alburni	1	2013, 2016	IT8050033 “Monti Alburni”	Cilento, Vallo di Diano e Alburni National Park	40.4705	15.4317	*Fagus* forest on non-acid soils	Unmanaged forest
Cilento	Monte Motola	1	2013, 2016	IT8050028 “Monte Motola”	Cilento, Vallo di Diano e Alburni National Park	40.3761	15.4694	*Fagus* forest on non-acid soils	Unmanaged forest
Aspromonte	Coppice stand	2	2017			38.1802	15.7843	Coppice and early-stage plantations	Agroforestry system
Aspromonte	Fruit orchard	1	2017			38.0602	15.7816	Coppice and early-stage plantations	Agroforestry system

**Table 3.** List of the datasets published on Zenodo.

Dataset name	doi	Dataset compiled by	Published papers
Annotated checklist of the beetles of Abeti Soprani, a silver fir forest of central Italy	10.5281/zenodo.3787025	Campanaro et al. (2020a)	Parisi et al. (2016) Parisi et al. (2020b)
Annotated checklist of the beetles of three beech forests in Gran Sasso National Park, central Italy	10.5281/zenodo.4310155	Campanaro et al. (2020b)	Zanetti and Parisi (2019) Sabatini et al. (2016) Parisi et al. (2021)
Annotated checklist of the beetles of three beech forests in Cilento, Vallo di Diano e Alburni National Park, southern Italy	10.5281/zenodo.4302220	Campanaro et al. (2020f)	Zanetti and Parisi (2019) Sabatini et al. (2016) Parisi et al. (2021)
Annotated checklist of the beetles of the broadleaved forest Bosco Pennataro, central Italy	10.5281/zenodo.4303930	Campanaro et al. (2020c)	Parisi et al. (2019) Parisi et al. (2020b)
Annotated checklist of the beetles of beech forests in Matese National Park, central Italy	10.5281/zenodo.4304401	Campanaro et al. (2020d)	Parisi et al. (2020a)
Annotated checklist of the beetles of chestnut agroforestry systems in Aspromonte, southern Italy	10.5281/zenodo.4304025	Campanaro et al. (2020e)	Parisi et al. (2020c) Toma and Parisi (2021)
